# Dependence of Relative Expression of NTR1 and EGFR on Cell Density and Extracellular pH in Human Pancreatic Cancer Cell Lines

**DOI:** 10.3390/cancers3010182

**Published:** 2011-01-04

**Authors:** Ulrike Olszewski-Hamilton, Gerhard Hamilton

**Affiliations:** 1 Cluster of Translational Oncology, Ludwig Boltzmann Society, c/o Balderichgasse 26A / 7-8, A-1170 Vienna, Austria; E-Mail: gerhard.hamilton@toc.lbg.ac.at; 2 Department of Surgery, Medical University of Vienna, Waehringer Guertel 18-20, A-1090 Vienna, Austria

**Keywords:** pancreatic cancer, neurotensin, epidermal growth factor receptor, extracellular acidosis, metastasis

## Abstract

Pancreatic adenocarcinoma is a devastating disease characterized by early dissemination and poor prognosis. These solid tumors express receptors for neuropeptides like neurotensin (NT) or epidermal growth factor (EGF) and exhibit acidic regions when grown beyond a certain size. We previously demonstrated increases in intracellular Ca^2+^ levels, intracellular pH and interleukin-8 (IL-8) secretion in BxPC-3 and PANC-1 pancreatic cancer cells in response to a stable NT analog. The present study aimed at investigation of the dependence of the relative expression of NT receptor 1 (NTR1) and EGFR in BxPC-3 and MIA PaCa-2 cells on cell density and extracellular pH (pH_e_). MTT assays revealed the NTR1 inhibitor SR 142948-sensitive Lys^8^-ψ-Lys^9^NT (8–13)-induced proliferation in BxPC-3 and PANC-1 cells. Confluent cultures of BxPC3 and HT-29 lines exhibited highest expression of NTR1 and lowest of EGFR and expression of NTR1 was maximal at slightly acidic pH_e_. IL-8 production was stimulated by Lys^8^-ψ-Lys^9^NT (8–13) and even enhanced at both acidic and alkaline pH_e_ in BxPC-3 and PANC-1 cells. In conclusion, our *in vitro* study suggests that one contributing factor to the minor responses obtained with EGFR-directed therapy may be downregulation of this receptor in tumor cell aggregates, possibly resulting in acquisition of a more aggressive phenotype via other growth factor receptors like NTR1.

## 1. Introduction

Adenocarcinoma of the pancreas is characterized by highly aggressive tumor growth and early metastasis resulting in a dismal prognosis for affected patients. Neuropeptides like bombesin, cholecystokinin, neuromedin N, neurotensin (NT), gastrin-releasing peptide and pituitary adenylate cyclase activating peptide stimulate growth and/or other cellular functions in various tumor types, including pancreatic cancer [1]. Production of NT and expression of NT receptor 1 (NTR1) were described for up to 90% of pancreatic adenocarcinomas and derived cell lines [2,3]. Ligand-binding to NTR1 stimulates cellular signal transduction through activation of phospholipase C-β (PLC-β), which generates the second messengers inositol 1,4,5-trisphosphate (IP3) and diacylglycerol (DAG) from phosphatidylinositol 4,5-bisphosphate [4,5]. IP3 triggers rapid release of Ca^2+^ from endoplasmic reticulum stores leading to transient elevation of cytosolic Ca^2+^. DAG activates protein kinase C (PKC) which plays a pivotal role as effector of the mitogen-activated protein kinase (MAPK) cascade, resulting in phosphorylation and activation of extracellular signal-regulated kinase 1 and 2 (ERK1/2). Activated receptor tyrosine kinases like epidermal growth factor receptor (EGFR) can furthermore interconnect to the Ras–Raf–MAPK pathway. In this case, signaling may be impaired by tyrosine kinase inhibitors.

On the other hand, EGFR was found to be expressed in 69% of cases of pancreatic cancer, and high expression was weakly associated with longer survival [6]. EGFR (also known as ErbB1) is a 170 kDa protein that belongs to the four-member ErbB family of transmembrane tyrosine kinase growth factor receptors. Monoclonal antibodies for anti-EGFR therapy, binding to the extracellular ligand-binding region of the receptor, inhibit dimerization and activation of the signaling cascade. However, two randomized trials revealed no significant survival benefit from combination of cetuximab with cytotoxic chemotherapy for advanced pancreatic cancer [7]. Small molecule inhibitors of EGFR inhibit autophosphorylation of the receptor and downstream signaling [8]. A minor but significant survival benefit was reported for therapy with gemcitabine in combination with erlotinib, leading to an approval of this combination therapy for advanced pancreatic cancer.

Our study aimed to investigate potential further causes for the failure of EGFR-directed therapy in pancreatic cancer. For this purpose, we particularly analyzed the relative expression of NTR1 and EGFR in dependence of cell density and acidosis *in vitro.* We used a panel of pancreatic cancer cell lines and the colon carcinoma cell line HT-29, the original source for cloning of NTR, for comparison of effects of NT-induced signal transduction [9]. NT analogs proved to trigger NTR1 antagonist SR 142948-sensitive elevation of intracellular Ca^2^+ levels in BxPC-3, PANC-1, HT-29 and, to a lower extent, MIA PaCa-2 cells [4,5,10,11]. Moreover, we reported NT analog-induced, SR 142948-sensitive intracellular alkalinization as a result of increased H^+^ flux across the cell membrane through activation of the Na^+^/H^+^ exchanger NHE1 by mitogen-and stress-activated kinase 1-mediated phosphorylation [12]. SR 142948-sensitive interleukin-8 (IL-8) production in response to NT analogs was detected in pancreatic cancer cell lines. Thus, NT/NTR1 signal transduction pathways lead to activation of NHE1 with increased H^+^ efflux resulting in extracellular acidification. Solid tumors like pancreatic cancer grown beyond a certain size exhibit irregular vascularization and contain highly acidic regions, which promotes genetic instability and selection of a more malignant phenotype of the cancer cells [14,15]. Genome-wide gene expression analysis was performed to detect effects of the stable NT analog Lys^8^-ψ-Lys^9^NT (8–13) on gene expression of BxPC-3 cells (data not shown). Results indicated upregulation of genes involved in the regulation of NHE1, hypoxic response and glycolysis induced by Lys^8^-ψ-Lys^9^NT (8–13) even under normoxic conditions. Therefore, it is suggested that growth factors like NT may be implicated in the early progression of pancreatic cancer by local extracellular acidification and induction of an aerobic glycolytic phenotype with higher metastatic potential in small cell aggregates. Though EGFR is abundantly expressed in pancreatic cancer and would thus provide a usable drug target, approaches to inhibit tumor growth have failed so far in clinical trials [7]. Hence, we aimed to investigate the relative expression of NTR1 and EGFR of BxPC-3, PANC-1 and MIA PaCa-2 pancreatic cancer cells in dependence of cell density and extracellular pH (pH_e_), representing conditions in tumor aggregates with declining vascular support.

## 2. Results

### 2.1. Effect of Lys^8^-ψ-Lys^9^NT (8-13) on Growth of BxPC-3, PANC-1 and MIA PaCa-2 Pancreatic Cancer Cells and the NTR1-Expressing Colon Cancer Cell Line HT-29

**Figure 1.**
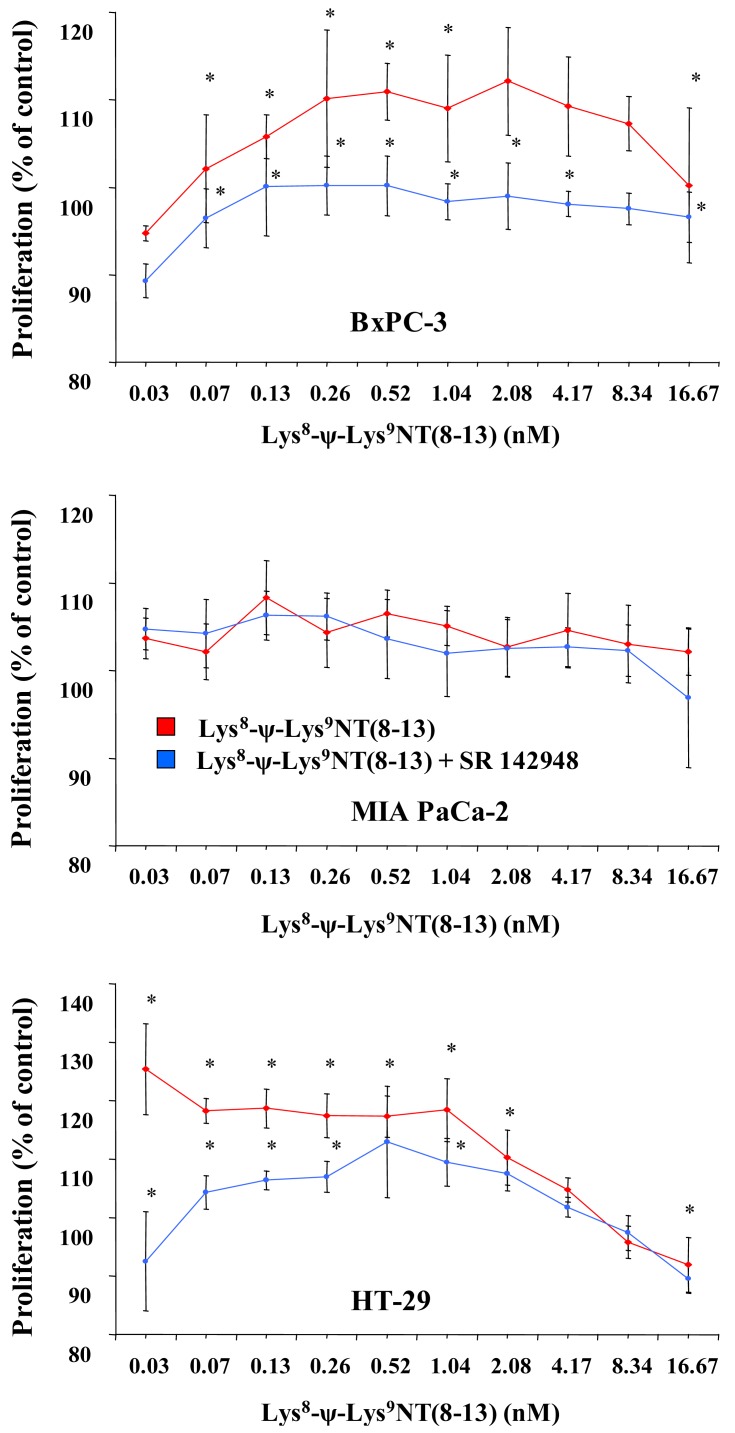
Proliferative responses of BxPC-3 (top), MIA PaCa-2 (middle) and HT-29 (bottom) cells to Lys^8^-ψ-Lys^9^NT(8–13) alone or in combination with 20 μM SR 142948 obtained by MTT proliferation assays after seven days of incubation. Data are presented as mean ± SD of duplicate measurements (differences to medium control and between NT- and NT/SR 142948-supplemented cultures: * p < 0.05).

Cell proliferation was assessed in MTT proliferation assays by incubation of cells with two-fold dilutions of Lys^8^-ψ-Lys^9^NT (8–13) at concentrations ranging from 0.07–16.67 nM under serum-free conditions for seven days. Dose-response curves for Lys^8^-ψ-Lys^9^NT (8-13) and BxPC-3, MIA PaCa-2 and HT-29 cells are shown in Figure 1. Proliferation of BxPC-3 cells was significantly stimulated by 0.07–1.04 nM Lys^8^-ψ-Lys^9^NT (8–13), whereas a concentration of 16.67 nM resulted in significant growth inhibition. Cell proliferation was not impaired by application of 20 μM SR 142948 in serum-free control medium alone, however, 20 μM SR 142948 in combination with Lys^8^-ψ-Lys^9^NT (8–13) revealed a dose-response relationship significantly different from NT-analog-stimulated growth over the whole concentration range, except at 8.3 nM Lys^8^-ψ-Lys^9^NT (8–13). Differences in the growth of MIA PaCa-2 cells treated with 0.07–16.67 nM Lys^8^-ψ-Lys^9^NT (8–13) and 20 μM SR 142948, either alone or in combination, were not significantly different from basal cell proliferation over the whole concentration range. NTR1-positive HT-29 colon cancer cells revealed increased growth at concentrations of 0.07–2.08 nM Lys^8^-ψ-Lys^9^NT (8–13); however, proliferation was significantly inhibited at 16.67 nM Lys^8^-ψ-Lys^9^NT (8–13), similar to BxPC-3 cells. The presence of 20 μM SR 142948 alone did not suppress proliferation below medium control levels in HT-29 cells; however, treatment with Lys^8^-ψ-Lys^9^NT (8–13) in combination with the antagonist impeded the cell growth at concentrations of 0.07–1.04 nM Lys^8^-ψ-Lys^9^NT (8–13) significantly.

Thus, application of Lys^8^-ψ-Lys^9^NT (8–13) exerted minor growth stimulatory effects at lower concentrations and inhibition of proliferation at highest concentrations in BxPC-3 and HT-29, but not in MIA PaCa-2 cells.

### 2.2. Dependence of Cell Cycle Distribution of Pancreatic Cancer Cell Lines and HT-29 Colon Carcinoma Cells on Cell Density

**Figure 2.**
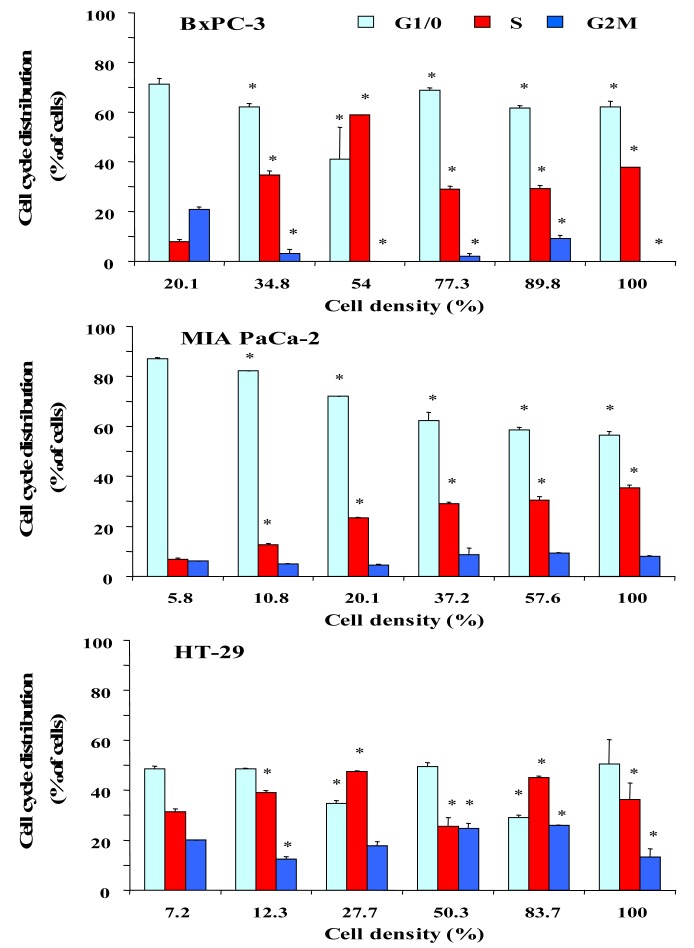
Dependence of the cell cycle distribution of BxPC-3 (top), MIA PaCa-2 (middle) and HT-29 (bottom) cells on the cell layer density. Data are presented as mean ± SD of duplicate measurements (differences in cell cycle phases to lowest density: * p < 0.05).

To investigate a putative correlation between cell cycle distribution and cell density, cells were seeded into six-well plates at increasing cell numbers and incubated in serum-supplemented culture medium until the cell layers in wells with highest cell numbers reached almost 100% of confluence. Figure 2 shows the dependence of the cell cycle distribution of BxPC-3, MIA PaCa-2 and HT-29 cells on the culture density. After an initial lag phase, BxPC-3 cells exhibited a considerable increase of cells in S phase with a maximum of DNA synthesis at 54% of confluence, followed by an increase of quiescent cells at high densities. MIA PaCa-2 cells, growing as scattered single cells, showed a large S phase fraction in highly dense cultures. Similar to BxPC-3 cells, proliferation of HT-29 cells was low during the lag phase and reached a maximum at approximately 28%, followed by a decrease of S phase cells in dense monolayers. According to these results, the different cell lines seem to become partially arrested at the S/G2M transition.

### 2.3. Dependence of NTR1 and EGFR Expression in Pancreatic Cancer Cell Lines and HT-29 Colon Carcinoma Cells on Cell Density

**Figure 3.**
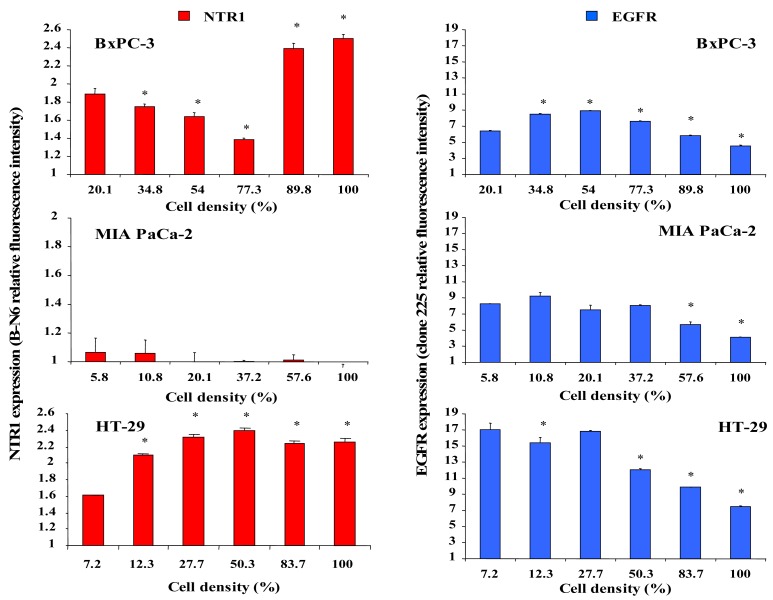
NTR1(left) and EGFR (right) expression of BxPC-3 (top), MIA PaCa-2 (middle) and HT-29 (bottom) cells in dependence of cell layer density determined by flow cytometry. Data are presented as mean ± SD of duplicate measurements (differences in expression to lowest density: * p < 0.05).

The effect of different cell densities on NTR1 cell surface expression was studied by flow cytometry using the monoclonal antibody B-N6, and results are shown in Figure 3. Cell surface expression of NTR1 in BxPC-3 cells decreased significantly with increasing cell density, but reached a maximum in cultures exceeding 77% of confluence (Figure 3, left, top). By contrast, MIA PaCa-2 cells exhibited low overall levels of NTR1 independent of cell density (Figure 3, left, middle). NTR1 levels of HT-29 cells were maximal at densities exceeding approximately 28 % (Figure 3, left, bottom).

**Figure 4.**
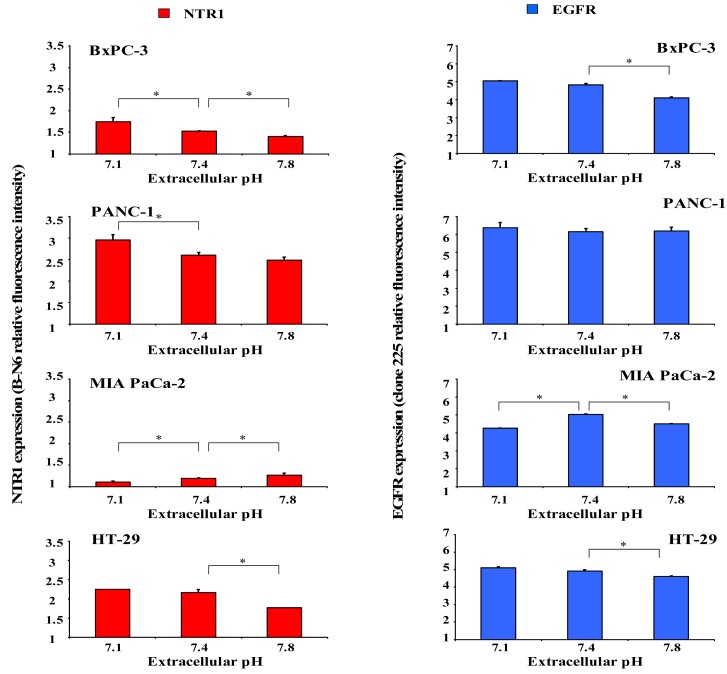
Dependence of NTR1 (left) and EGFR (right) expression in BxPC-3, PANC-1 and MIA PaCa-2 cells on pH_e_, determined by flow cytometry. Data are presented as mean ± SD of duplicate measurements (differences to physiological pH 7.4: * p < 0.05).

For comparison, cell surface expression of the other growth factor receptor EGFR was quantified in dependence of cell density by use of the monoclonal antibody clone 225. In general, EGFR revealed higher expression levels than NTR1 in all cell lines (Figure 4, right). BxPC-3 cells exhibited augmented expression of EGFR at densities up to 77% and significantly reduced EGFR levels near confluence (Figure 3, right, top). EGFR expression in MIA PaCa-2 cells was significantly decreased above 37% of confluence (Figure 3, right, middle). HT-29 cells revealed considerable EGFR levels in scattered cells and reduced expression in cultures at and above 50% of confluence (Figure 3, right, bottom).

### 2.4. Dependence of NTR1 and EGFR Expression in Pancreatic Cancer Cell Lines and HT-29 Colon Carcinoma Cells on pH_e_

Since acidic regions are frequently found within solid tumors, it was investigated whether distinct extracellular pH conditions altered membrane expression of NTR1 and EGFR. For this purpose, cells were incubated in serum-free media of pH 7.1, 7.4 or 7.8 for four days and receptor levels analyzed by flow cytometry. As demonstrated in Figure 4 (left), BxPC-3 and PANC-1 cells revealed significant upregulation of NTR1 expression at pH_e_ 7.1 and downregulation at pH_e_ 7.8, in comparison to pH_e_ 7.4. Treatment with 10 nM Lys^8^-ψ-Lys^9^NT (8-13) resulted in significant internalization of NTR1 in BxPC-3 and PANC-1 cells independent of pH_e_ (data not shown). On the contrary, the low NTR1 expression in MIA PaCa-2 cells declined with decreasing pH_e_ and the presence of Lys^8^-ψ-Lys^9^NT (8–13) did not alter NTR1 expression significantly.

Parallel experiments to evaluate a putative dependence of EGFR expression on pH_e_ were performed for comparison (Figure 4, right). BxPC-3 cells exhibited high levels of EGFR that were significantly decreased at pH_e_ 7.8; however, receptor expression was not altered in PANC-1 cells.

Again, MIA PaCa-2 cells revealed effects opposite to those of BxPC-3 and PANC-1 cells with downregulation of EGFR at acidic and alkaline pH_e_, respectively.

Similar to BxPC-3 and PANC-1 cells, NTR1 levels in HT-29 cells decreased with increasing pH_e_ and expression of the receptor was considerably reduced in the presence of the NT analog, independent of pH_e_. EGFR expression was highest under physiological conditions and decreased at acidic and alkaline pH_e_ in Lys^8^-ψ-Lys^9^NT (8-13)-treated cells (data not shown).

**Figure 5.**
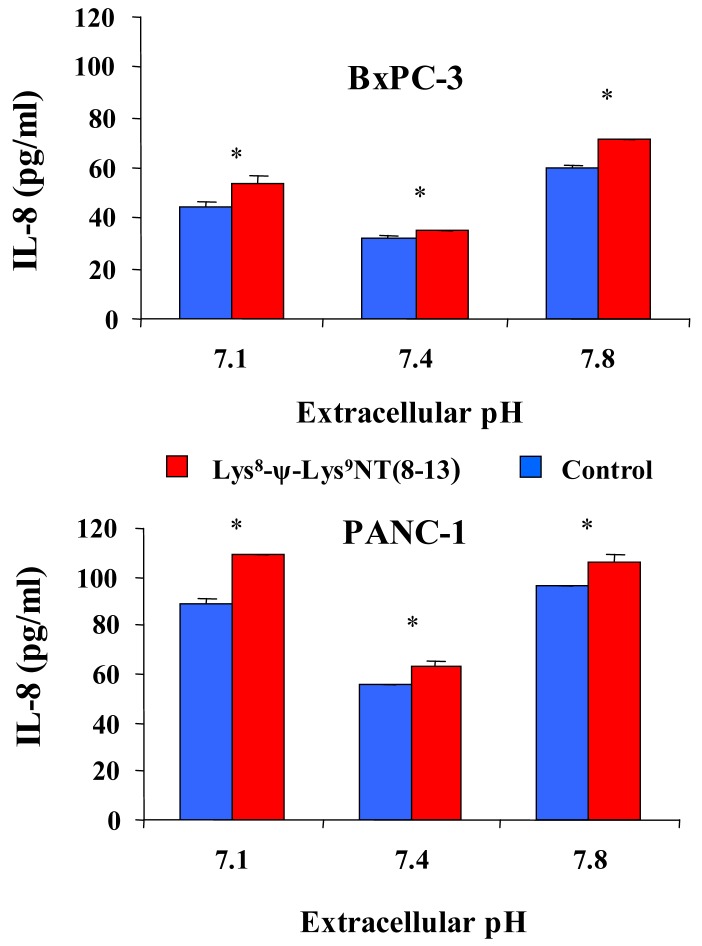
Concentrations of IL-8 in culture supernatants of untreated or Lys8-ψ-Lys9NT (8-13)-treated BxPC-3 (top) and PANC-1 (bottom) cells in dependence of different extracellular pH conditions. Data are presented as mean ± SD of duplicate measurements (differences between medium and NT-supplemented samples: * p < 0.05).

In addition, effects of acidic or alkaline extracellular conditions on constitutive and Lys^8^-ψ-Lys^9^NT (8-13)-induced IL-8 production were investigated by stimulation of cells with 100 nM NT-analog in serum-free media of pH 7.1, 7.4 or 7.8 for seven hours. As shown in Figure 5 for BxPC-3 and PANC-1 cells, constitutive as well as NT analog-induced secretion of IL-8 was significantly elevated under acidic and alkaline conditions. The proportion of IL-8 that was enhanced by Lys^8^-ψ-Lys^9^NT (8-13) was higher under acidic and alkaline conditions compared to pH_e_ 7.4. BxPC-3 cells exhibited an increase of NT-analog-induced IL-8 of +20.4 ± 8.5% under acidic and 19.7 ± 4.0% under alkaline conditions, while the fraction of NT-analog-induced IL-8 production amounted to +9.7 ± 3.5% under physiological conditions. PANC-1 cells revealed increases of +22.2 ± 2.5% and +10.8 ± 3.7% at pH_e_ 7.1 and 7.8, respectively, in response to Lys^8^-ψ-Lys^9^NT (8–13) compared to ΔIL-8 of +13.1 ± 4.8% at physiological pH_e_.

### 2.5. Discussion

Pancreatic adenocarcinoma is characterized by highly aggressive tumor growth and early metastasis, resulting in a dismal prognosis for affected patients. EGF and neuropeptides like bombesin, cholecystokinin, neuromedin N and NT may act as growth factors and stimulate proliferation on the one hand and/or affect other cellular functions in different tumor cell types, including pancreatic cancer, on the other hand [1,15]. Expression of EGFR was reported in approximately 70% and production of NT and expression of NTRs in up to 90% of pancreatic tumors and derived cell lines [2,3,6]. Despite their abundant presence in cancer cells, suggesting them as useful drug targets, approaches to suppress EGFR- or NTR1-mediated signal transduction pathways by use of anti-EGFR monoclonal antibodies, such as cetuximab, small molecule tyrosine kinase inhibitors like erlotinib or the nonpeptide NTR1 antagonist SR 48692, have not met expectations in clinical studies so far [7]. This has been attributed to limited accessibility of the tumor cells for the large monoclonal antibodies. Furthermore, *KRAS* mutations leading to persistent activation of cellular signaling events downstream of EGFR are found in 50–95% of pancreatic cancer samples and render proliferation of tumor cells independent of EGFR [7,16]. However, application of gemcitabine combined with erlotinib for first-line treatment of advanced pancreatic cancer was approved by the U.S. Food and Drug Administration in 2005 [17].

Initial evidence of the role of the NT-NTR system in various tumor entities concentrated attention to development of NTR inhibitors that were demonstrated to be partially effective in inhibiting basal and, preferentially, NT-stimulated growth of tumors in experimental animal models [3]. In particular, the selective nonpeptide NTR1 antagonist SR 48692 was tested in a double-blind, randomized, phase II-III maintenance study *versus* placebo in patients with extensive stage small cell lung cancer following a first-line chemotherapy with cisplatin and etoposide (NCI identifier NCT00290953) and revealed only sporadic responses at best, which resulted in discontinuation of the development as an anticancer drug (Evaluation of the Overall Survival of Meclinertant *Versus* Placebo After a First Line Chemotherapy With Cisplatin + Etoposide; Clinical Trials, 2009) [18]. This failure to suppress tumor cell proliferation may be explained by the capability of cancer cells to employ paracrine/autocrine growth factors other than NT for proliferation and/or by the significance of NT-NTR signaling for tumor-associated cellular functions besides growth control, for example the acidification of extracellular compartment [13].

**Figure 6.**
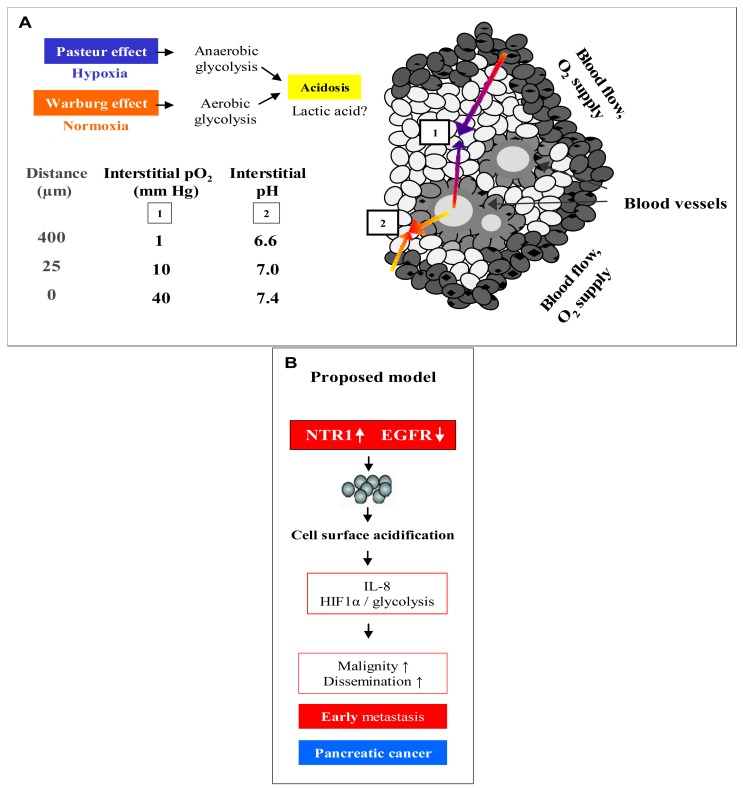
Proposed model of a mechanism that may be involved in metastasis and dissemination of pancreatic cancer already at an early stage of the disease. (**A**) Overview of the mechanisms leading to extracellular acidosis commonly found in solid tumors of larger size, and (**B**) proposed model for extracellular acidosis.

Figure 6A demonstrates an overview of the mechanisms leading to extracellular acidosis commonly found in solid tumors of larger size. Low interstitial pH may be either due to insufficient supply of the tumor with nutrients and oxygen as a result of irregular vascularization. Decrease of the partial pressure of oxygen (pO2) with increasing distance from blood vessels leads to a metabolic switch of hypoxic tumor cells from respiration to anaerobic glycolysis with extracellular accumulation of lactic acid (Pasteur effect) [19]. Moreover, cancer cells draw energy from anaerobic glycolysis even under normoxic conditions (Warburg effect) [20,21]. According to our own results, we propose a model for extracellular acidosis, which is known to increase the metastatic potential of tumor cells and promotes dissemination responsible for poor clinical outcome involving NT (Figure 6B). In contrast to NTR1, EGFR seems to become downregulated in the tumor cell aggregates during their switch to metastasis.

Own experiments revealed expression of NTR1 and NTR3 at the mRNA level in all three pancreatic cancer cell lines and cell surface expression of NTR1 in BxPC-3, PANC-1 and MIA PaCa-2 pancreatic cancer cells was demonstrated using the monoclonal antibody B-N6 directed to NTR1. In the present study, proliferative responses of the cell lines to the NT analog were tested in MTT assays. Growth of BxPC-3 and HT-29 cells was stimulated to a minor degree at lower concentrations of Lys^8^-ψ-Lys^9^NT (8–13) in an SR 142948-sensitive manner and was inhibited at higher concentrations, contrary to MIA PaCa-2 cells lacking a specific growth response.

In order to assess the relationship between NTR1 and cell density, membrane expression of the receptor was determined in cultures of varying cell numbers. According to these results, the cell lines studied here seem to become arrested in the S/G2M phases upon reaching confluence. NTR1 expression varied with the cell density and was found to be maximal at medium density in HT-29 cells and at confluence in BxPC-3, respectively, whereas MIA PaCa-2 cells exhibited lower NTR1 expression in general, independent of cell density. Since the relative significance of EGFR and NTR1 in regard to tumor cell proliferation and dissemination has not been elucidated for the pancreatic cancer cell lines so far, we compared the dependence of the expression of both receptors on cell density and pH_e_. The ubiquitous EGFR exhibited significantly decreased cell surface expression at cell densities near confluence in BxPC-3 and MIA PaCa-2 pancreatic cancer as well as HT-29 colon cancer cells, in contrast to NTR1. These findings do not correlate with the *KRAS* status of the cell lines, since BxPC-3 and HT-29 are wildtype, whereas MIA PaCa-2 has mutated *KRAS* (G12C) [22]. Anti-EGFR treatment is expected to have no effect in MIA PaCa-2 and PANC-1 cells characterized by overactivated mutant *KRAS.* Treatment of cells with 10 nM Lys^8^-ψ-Lys^9^NT (8–13) did not modulate EGFR in BxPC-3 and PANC-1 cells (data not shown). The results are in good agreement with experiments which described cell density-dependent inhibition of EGFR signaling by p38α MAPK [23]. Confluent activation of p38α MAPK inhibits EGFR-induced proliferation through receptor degradation and may be enhanced further by NT-induced stimulation of the same kinase in NT-producing cell lines like BxPC-3 and HT-29 [11,24]. Data point to a significant function of NTR1 in biological processes of non-dividing, resting pancreatic cancer cells, corroborating the conflicting experimental and clinical reports on the role of NT as a growth factor and the inability of the NTR1 inhibitor SR 48692 to impede tumor progression in patients.

Solid tumors of larger size with irregular vascularization are known to contain highly acidic regions with pH < 6.8 [20,25,26]. Previously, we had demonstrated for the first time that NT induced NHE1-dependent intracellular alkalinization and extracellular acidification mediated by mitogen- and stress-activated kinase 1/2 (MSK1/2) in pancreatic cancer cells [11]. Therefore, the pancreatic cell lines were investigated using media adjusted to different pH values. Normally, cancer cells exhibit reduced growth under acidic conditions; however, all three pancreatic cancer cell lines proliferated more rapidly when incubated in medium of pH 7.1, while cell numbers were markedly decreased at an alkaline pH of 7.8. Both BxPC-3 and PANC-1 cells revealed enhanced NTR1 cell surface expression under acidic extracellular conditions, unlike MIA PaCa-2 cells exhibiting NTR1 levels increasing with pH_e_. EGFR expression was not significantly altered in dependence of pH_e_, with the exception of slightly decreased receptor levels in BxPC-3 cells at alkaline and in MIA PaCa-2 cells at acidic and alkaline pH_e_ values.

The main source of intratumoral acidity has been considered to be lactate produced during anaerobic or aerobic glycolysis, respectively [27,28]. To define further targets of NT in pancreatic cancer cells, whole-genome gene expression analysis was performed using Lys^8^-ψ-Lys^9^NT (8–13)-treated BxPC-3 cells and, most interestingly, induction of hypoxia-inducible factor 1α (HIF-1α) and upregulation of genes implicated in glycolytic metabolism was observed although cells were incubated under normoxic conditions.

In conclusion, NT/NTR1 signaling in pancreatic cancer cells seems to promote the induction of a metastatic phenotype, prior to acidosis resulting from a metabolic switch to glycolysis common for larger cancers, in contrast to its varying effects on tumor cell proliferation. Localized acidic extracellular conditions favor tumor cell invasion at a very early stage of tumor development [29]. This is consistent with the literature of the contribution of acidic pH_e_ to tumor invasion through selection of aggressive tumor cells, suppression of apoptosis, induction of proinvasive mediators and stimulation of protease secretion [30,31]. A central player of metastasis is IL-8, an autocrine/paracrine factor that affects several cellular processes and, importantly, triggers release of matrix metalloproteinases or urinary plasminogen activator from cells [32]. Upregulation of IL-8 in solid tumors like colon and pancreatic cancer is known to be stimulated by the prevalent hypoxic and acidic environment [32,33]. Own experiments clearly demonstrated that Lys^8^-ψ-Lys^9^NT (8–13) induced secretion of IL-8 in BxPC-3 and PANC-1 cells. These findings, taken together, strongly indicate that NT signaling in pancreatic cancer cells can initiate and/or stimulate cellular processes which contribute to the transformation of cells holding less aggressive potential to more metastatic cells. Downregulation of EGFR at higher cell densities concomitant with upregulation of NTR1 seems to indicate mutual exclusive roles for these receptors. EGFR may be important in the initial growth of pancreatic tumor cells and replaced by increased expression of other growth factor receptors, like NTR1, during metastatic dissemination.

## 3. Experimental Section

### 3.1. Chemicals and Solutions

Unless otherwise noted, all chemicals and solutions were obtained from Sigma-Aldrich (St. Louis, MO, U.S.). The stable NT analog Lys^8^-ψ-Lys^9^NT (8–13) (H-Lys^8^-ψ-Lys^9^-Pro^10^-Tyr^11^-Ile^12^-Leu^13^-OH) was purchased from Bachem (Weil am Rhein, Germany), dissolved in distilled water at a concentration of 5 mg/mL (6.7 mM Lys^8^-ψ-Lys^9^NT(8–13)) and aliquots stored at −20 °C. SR 142948 from Tocris Cookson (Bristol, U.K.) was dissolved in dimethyl sulfoxide (DMSO) at 5 mM. Aliquots were stored at −20 °C. Ca^2+^- and Mg^2+^-free Dulbecco's phosphate buffered saline (PBS) for trypsinization of cell cultures was purchased from Gibco/Invitrogen (Carlsbad, CA, U.S.).

### 3.2. Cell Culture

The pancreatic cancer cell lines BxPC-3, MIA PaCa-2 and PANC-1 and the colon carcinoma cell line HT-29 were obtained from the American Type Culture Collection (ATCC; Rockville, MD, U.S.) and cultivated in RPMI-1640 bicarbonate medium (Seromed, Berlin, Germany) supplemented with 10% heat-inactivated fetal bovine serum (Seromed), 4.0 mM L-glutamine, 50 units/mL penicillin, 50 μg/mL streptomycin and 100 μg/mL neomycin in a humidified incubator (Heraeus Cytoperm, Hanau, Germany; 5% CO_2_, 37 °C, 95% humidity). Viability of cells assessed by trypan blue exclusion was > 95% for all experiments. Cell culture media yielding pH values of 7.1, 7.4 and 7.8 under the tissue culture conditions (5% CO_2_) were prepared by supplementation of bicarbonate-free RPMI-1640 medium with 10, 25 and 45 mM NaHCO_3_, respectively. Cells were subcultivated by trypsinization with 0.05% trypsin/0.02% ethylene diamine tetraacetic acid (EDTA) three times a week and checked for mycoplasma contamination (Mycoplasma PCR ELISA, Roche Diagnostics, Vienna, Austria).

### 3.3. Cell Proliferation Assay

In order to determine the effects of test compounds on cell proliferation, 6–10 serial two-fold dilutions of the compounds were pipetted into 96-well microtiter plates (Greiner, Kremsmuenster, Austria) at a volume of 100 μL medium per well followed by addition of 0.5 × 10^4^ cells at another 100 μL medium/well. Tests were performed in triplicate. Microtiter plates were incubated under tissue culture conditions and cell proliferation measured using a modified MTT (3-(4, 5-dimethylthiazol-2-yl)-2,5-diphenyltetrazoliumbromide) assay (EZ4U, Biomedica, Vienna, Austria) according to the manufacturer's instructions. Optical densities were measured at 450 nm using the EL × 800 microplate reader (BioTek, Bad Friedrichshall, Germany). Proliferation of each cell line in the absence of test substances was determined using separate wells and set to 100% for calculation.

### 3.4. Flow Cytometry

4 × 10^5^ native cells were incubated with primary mouse monoclonal antibodies clone B-N6 (Cell Sciences, Canton, MA, U.S.) or clone 225 (Abcam, Cambridge, U.K.) in medium in microtiter plates (Greiner, Kremsmuenster, Austria) at 4 °C overnight for detection of NTR1 and EGFR, respectively. Cells were then washed with PBS, stained with goat anti-mouse fluorescein isothiocyanate (FITC)-labeled IgG secondary antibody (dilution 1:50, 4 °C, 20 min), washed again and analyzed using the Cytomics FC500 flow cytometer (Beckman Coulter, Fullerton, CA, U.S.) acquiring 5,000 cells per run. Antigen expression was calculated as relative fluorescence intensity = mean fluorescence antibody/mean fluorescence control.

### 3.5. Cell Cycle Analysis

5 × 10^5^ cells per well cultured in 6-well plates (Greiner) were trypsinized, washed with PBS, fixed in 70% ethanol at −20 °C for 30 min, washed again, resuspended in staining solution (PBS containing 20 μg/mL propidium iodide, 5 μg/mL ribonuclease A and 0.05% Nonidet P-40) and incubated at room temperature overnight. Washed cells were analyzed by flow cytometry (Cytomics FC500, Beckmann Coulter) acquiring 1 × 10^4^ cells per run. MultiCycle AV software (Phoenix Flow Systems, San Diego, CA, U.S.) was employed for calculation of cell cycle distribution from linear histograms. Percentages of cells in cell cycle phases G1/0 (resting), S (DNA synthesis) and G2M (mitotic cells) were recorded.

### 3.6. Determination of IL-8

Cellular IL-8 production was detected in cell culture supernatants using the Strakine Tool Box ELISA Kit (Strathmann Biotec, Hamburg, Germany) according to the manufacturer's instructions.

### 3.7. Statistics

All experiments were performed at least in duplicate and calculations were carried out using Origin Scientific Graphing and Analysis Software (Northampton, MA, U.S.) and Microsoft Excel (Redmond, CA, U.S.). Values are shown as mean ± standard deviation (SD). Statistical analysis was done using Student's t-test assuming normal distribution for the experimental measurements. Differences with * p < 0.05 were regarded as statistically significant.

## 4. Conclusions

In conclusion, NT-NTR signaling in pancreatic cancer cells seems to promote the induction of a metastatic phenotype by activation of distinct stress-activated signal transduction pathways, generation of intracellular alkalinization/extracellular acidification, increased expression of IL-8 and possibly HIF-1α, in contrast to its varying effects on tumor cell proliferation. Growth factors like NT seem to have the capability to stimulate NHE1 and to promote an acidic extracellular microenvironment favoring tumor cell invasion at a very early stage of tumor development, prior to acidosis resulting from a metabolic switch to glycolysis in larger cancers. In contrast to the expression of NTR1, EGFR is downregulated in cell cultures of increasing density, possibly indicating a minor role of this receptor in the cellular switch to a highly metastatic phenotype. These findings may help to explain the early tumor dissemination and the minor effects of an EGFR-directed therapy common for pancreatic cancer patients.
